# The expression profiling of serum miR-92a, miR-134 and miR-375 in acute ischemic stroke

**DOI:** 10.2144/fsoa-2022-0074

**Published:** 2023-02-20

**Authors:** Aya Tarek Salman, Olfat Shaker, Shereen Saeid Elshaer, Ahmed Elshafei

**Affiliations:** 1 Department of Biochemistry, Faculty of Pharmacy, Egyptian Russian University, Cairo, 11829, Egypt; 2 Department of Biochemistry & Molecular Biology, Faculty of Medicine, Cairo University, Cairo, 12613, Egypt; 3 Department of Biochemistry & Molecular Biology, Faculty of Pharmacy (Girls), Al-Azhar University, Cairo, 11884, Egypt; 4 Department of Biochemistry, Faculty of Pharmacy, Heliopolis University, Cairo, 11785, Egypt; 5 Department of Biochemistry & Molecular Biology, Faculty of Pharmacy (Boys), Al-Azhar University, Cairo, 11884, Egypt

**Keywords:** AIS, biomarkers, miR-134, miR-375, miR-92a, miRNA

## Abstract

**Aim:**

To investigate the expression profile and diagnostic potentials of serum miR-92a, 134, and 375 in acute ischemic stroke (AIS) patients.

**Materials & methods:**

Serum miRs-92a, 134, and 375 expression profiles were estimated by qRT-PCR for 70 AIS patients, age-matched with 25 control subjects. Their diagnostic potential was estimated by ROC analysis.

**Results:**

Down-expression of miR-92a and miR-375 was found (56; 96.5%; -1.86 ± 1.36; and 53; 91.4%; -1.63 ± 1.38, respectively), while miR-134 showed a predominant upregulation (46; 79.3%; 0.853 ± 1.34). The diagnostic accuracy was the highest for miR-92a and miR-375 (area under the curve = 0.9183 and 0.898, respectively), with greater specificity for miR-375 (Sp = 96%).

**Conclusion:**

Serum miR-92a and miR-375 could be promising early detective biomarkers of AIS.

## Plain language summary

This study aimed to examine how miR-92a, 134, and 375 in acute ischemic stroke (AIS) patients were expressed and if they could be used to diagnose the disease. Hence, their expression profiles were assessed in the serum of 70 AIS patients and 25 controls. Results showed that miR-92a and miR-375 were downregulated, while miR-134 was mostly upregulated. miR-92a and miR-375 had the best diagnostic accuracy, but miR-375 was more specific. Therefore, miR-92a and miR-375 show promise as potential early AIS biomarkers.

A stroke is a sudden life-threatening disease concerning a fall in brain functions, with an unclear outcome, especially for the chances of recovery [1]. Globally, stroke is the 2nd leading death cause with subsequent disabilities [2]. Stroke is a major mortality cause in Egypt, where the incidence rate is 137,000 to 250,000 yearly [3].

The majority of stroke cases are acute ischemic stroke (AIS), representing 87% of all stroke cases [4]. Earlier diagnosis of stroke and immediate treatment can reduce the damage and disability of the brain for the affected individual and successful rehabilitation [5].

Urgent contrast-MRI brain imaging is an essential first step in managing patients with stroke [6,7]. However, a novel non-invasive biological biomarker with proper sensitivity and specificity is needed for the early identification of AIS [8].

MicroRNAs (miRNAs) are a group of small non-coding RNAs of 18–25 nucleotides that have been shown to modulate protein expression at the post-transcriptional level since their discovery in *Caenorhabditis elegans* in 1993 [9].

Recently, miRNAs have captured the attention of different aspects of clinical research concerning their origin and functions in various human pathological states, including stroke. They are considered promising biomarkers due to their proper specificity and sensitivity in many diseases, stability in circulation in addition to their circulating accessibility besides the ease of biochemical analysis [10,11]. Therefore, they get attention for being non-invasive stable biomarkers for most diseases [12,13].

MicroRNA-92a is a putative oncogene. It has been reported as a key regulator and diagnostic biomarker that participates in different types of cancer where it can regulate tumorigenesis and metastasis [14–16]. Besides its thoroughly investigated role in cancers, miR-92a showed a regulatory role in the pathogenesis of atherosclerotic vascular diseases because of its participation in cholesterol homeostasis, endothelial function, vascular inflammation, platelet production as well as aggregation, and blood pressure regulation [17–19]. Moreover, miR-92a was found to induce angiogenesis in ischemic mice [20]. Recently, a study reported that miR-92a expression could have an important role in atherosclerosis and hypertension which may influence ischemic stroke [21]. Based on these studies, miR-92a was selected to be included in this study to assess for the 1st time its clinical value in AIS.

MicroRNA-375 has been discovered in 2004 as pancreatic tissue-specific miRNA regulating β-cells and insulin secretions [22]. Many studies found that miR-375 acts as a tumor suppressor in malignant cells [13,23], also it has a pathological role in heart diseases like cardiac hypertrophy, myocardial infarction, and heart failure, and a direct relation with insulin secretion in diabetic patients [24,25]. Due to its cardiometabolic involvements, miR-375 was included to find its diagnostic potential in AIS.

The apoptotic effect of miR-134 was reported in experimental models of ischemic stroke, but without being investigated in the patient's circulation [26].

The current study aimed to examine for the first time the expression pattern and the diagnostic potential of miRNAs 92a, 375 & 134 in AIS patients' sera as non-invasive molecular markers.

## Materials & methods

### Study participants

This study was a pilot observational retrospective study enrolled 95 adult males, 70 of them were AIS patients admitted to the emergency department of Kasr El-Ainy hospital, Cairo University, Egypt, with stroke-suggestive symptoms, and their diagnosis was confirmed by contrast MRI performed by a 3.0 Tesla whole body imaging system with an augmented clinical decision, while the other 25 subjects were apparently healthy control subjects. Patients with a history of stroke, myocardial infarction, intracranial hemorrhage, peripheral vascular disease, and neuropsychological disorders were excluded. The other 25 subjects were age-matched healthy controls selected from the outpatient clinic of Kasr El-Ainy hospital, Cairo University, Egypt. The protocol of this study was approved by the ethical committee of Kasr El-Ainy hospital and the ethics committee of the Faculty of Pharmacy (Girls) at Al-Azhar University in Cairo (no. 157). Written informed consent was obtained from all enrolled subjects or the corresponding relatives of AIS patients.

### Sampling & methodology

The BD vacutainer system was used for the withdrawal of 5 mL of peripheral venous blood samples from all subjects. Serum separator tubes were used for serum separation, where blood was left for 15 min to clot, and then centrifuged for 10 min at 4000 r.p.m. The isolated sera were stored at -80°C until being analyzed.

### Total serum RNA isolation, including microRNAs

Isolation of total RNA from serum samples was performed using the miRNeasy Mini Kit (cat. no. 217004; Qiagen, Germany), which contained lysis reagent (phenol/guanidine thiocyanate) and silica membrane-based purification of total RNA. The extraction started with the addition of a 200 μl serum sample to 1000 μl of Qiazol lysis reagent at room temperature for 5 min.

Then 200 μl of chloroform was added into the denaturized serum resulting in the separation of the lysate into organic and aqueous phases. The tubes were well mixed by vortexing for 15 s followed by centrifugation for 15 min at 14000 r.p.m. at 4°C. 900 μl of ethanol was added to the extracted aqueous layer (~600 μl) and mixed by pipetting up and down several times. 700 μl of the mix was applied into RNeasy mini spin columns and then centrifuged at 14000 r.p.m. for 15 s, the flow-through was then thrown away.

To wash the mini spin column, two buffer solutions (RWT and RPE) were used consecutively and then centrifuged at 14000 r.p.m. for 15 s at room temperature. To make sure the spin columns are free from ethanol and dry before elution, they were placed in 2 ml collecting tubes and centrifuged at 14000 r.p.m. for 2 min. Finally, to elute Silica-bound RNA, 50 μl of RNase-free water was added onto the mini spin column and then centrifuged at 14000 r.p.m. The eluted RNA was divided into two portions, 5 μl for NanoDrop spectrophotometer-based RNA quantitation and purity assessment, while the remainder was stored at -80°C to be used in the step of RNA reverse transcription.

### Purified RNA quantification, including miRNAs

Quantitation of RNA and purity assessment was performed by the NanoDrop^®^ (ND)-1000 spectrophotometer (NanoDrop Technologies, Inc., USA). The samples were measured by loading the NanoDrop-1000 with 1 μl of samples-extracted RNA and their readings were recorded and calculated according to Beer-Lambert's law. The concentration of the nucleic acid in the sample was measured at absorbance 260 nm (A260 = 1 = 44 μg/ml). The generally accepted ratios for 260/280 and 260/230 ratios were 1.9–2.1 and >1.7, respectively.

### Complementary DNA (cDNA) synthesis from the extracted miRNAs

This was done using miScript^®^ II RT kit (Qiagen, Germany, cat. no. 218161) as miScriptHiSpec Buffer was used for selective RT of mature miRNA into cDNA. The reverse transcriptase master mix was prepared on the ice of a total volume of 20 μl by using 4 μl miScriptHiSpec buffer (5×), 2 μl miScriptHiSpec buffer (10×), 2 μl miScript reverse transcriptase mix, 7 μl RNase free water, 5 μl RNA template (Samples-extracted RNA) in addition to 5 μl RNA template (Samples-extracted RNA). The final cDNA of each sample was stored undiluted at -80°C to be used in the next step.

### Mature miRNA quantification using qRT-PCR

In this step, mature miRNA quantitative detection was done through a protocol using miScript SYBER^®^ Green PCR kit (Qiagen, cat. no. 218073). Hs_miRNA-92a, Hs_miRNA-134, and Hs_miRNA-375 are the target-specific primers assay (forward primers) used for the selected miRNAs in addition to the housekeeping gene (internal control) Hs_SNORD68, in this step.

Firstly, the stored cDNA samples,miScript SYBR Green PCR, and miScript Primer Assay kits were allowed to thaw at room temperature. 200 μl RNase-free water was added to cDNA samples for dilution. Then, the reaction mix of a total volume of 25 μl was prepared by using the following components 12.5 μl QuantiTect SYBR Green PCR Master Mix (2×), 2.5 μl miScript Universal primer (10×), 2.5 μl miScript Primer assay (10×), 5 μl RNase free water and 2.5 μl Template cDNA.

Rotor-Gene Q 72-well rotor (Qiagen, USA) was used for the quantification reaction followed by 40 amplification cycles where each cycle was done through 3 cycles which were programmed under certain conditions: incubation at 95°C for 15 min for the initial activation step followed by 3 steps cycling of DNA denaturation at 94°C for 15 s, annealing at 55°C for 30 s and extension for 70°C for 30 s.

### Results calculation

Melting curves were analyzed after the completion of qRT-PCR cycles to validate and confirm the targeted miRNAs' specific expression. Also, Calculations of cycle threshold (Ct) values were automatically calculated using Rotor-Gene Q software 2.1 (Qiagen).

Accordingly, the relative expression of SNORD-68 was evaluated using the ΔCt method by the subtraction of Ct values of SNORD-68 from Ct values of the targeted miRNAs and this was done for both control and patients' groups. Then, the calculation of ΔΔCt values was performed by subtracting ΔCt values of the control group from ΔCt values of the patients' group. Finally, the fold changes (FC) which is the expression ratio or relative quantitation (Rq) for the target miRNAs were calculated using the 2^-ΔΔCt^ method [27].

### Statistical analysis

The statistical analysis was performed, and charts were built using GraphPad Prism 8.02. The data were presented as mean ± standard deviation (SD), median (interquartile range (IQR)), number, and percentage. The Kolmogorov-Smirnov normality test was used to determine the normal distribution pattern between the data of the groups. The difference between two groups was compared by the Student's *t*-test, Man-Whitney U tests when appropriate, and the one-way analysis of variance (ANOVA) was used for the multiple comparisons between more than two groups.

The receiver operator characteristic (ROC) curve and the area under the curve (AUC) were used to determine the diagnostic accuracy and cutoff values for each miRNA. While Pearson's correlation test was used for the correlation between the expression level of miRNAs and the routine biochemical investigations of AIS patients. A two-sided p < 0.05 was considered statistically significant.

## Results

### Demographics description & routine biochemical findings

This study was carried out on 70 AIS male patients aged (59.4 ± 8.36 years) with suggestive symptoms and confirmed diagnosis by clinical decision and MRI findings and 25 male age-matched (57.3 ± 3.27 years) control subjects. The demographic and routine biochemical findings are listed in Table 1.

**Table 1.** Demographics description and routine biochemical findings.

n	25	70	–
Age (range, years)	49–62	42–79	0.2328
Mean ± SD, years	57.3 ± 3.27	59.4 ± 8.36	
RBG (range, mg/dl)	85.0–128	105–398	<0.0001^†^
Mean ± SD, mg/dl	103 ± 13.1	218 ± 98	
T.Ch (range, mg/dl)	134–202	137–259	0.0020^†^
Mean ± SD, mg/dl)	180 ± 18.4	196 ± 26.7	
TG (range, mg/dl)	125–168	52–219	0.1162
Mean ± SD, mg/dl	147 ± 15.5	132 ± 37.5	
LDL-ch (range, mg/dl)	78.0–142	72–178	0.0047^†^
Mean ± SD, mg/dl	108 ± 5.16	122 ± 23.7	
HDL-ch (range, mg/dl)	30.0–76.0	35–62	0.0411^†^
Mean ± SD, mg/dl	50.8 ± 12.8	47 ± 6.73	
VLDL-ch (range, mg/dl)	19.2–42.0	10.4–43.8	<0.0001^†^
Mean ± SD, mg/dl	32.2 ± 6.84	26.5 ± 7.49	
Cr. (range, mg/dl)	0.860–1.21	0.88–1.50	<0.0001^†^
Mean ± SD, mg/dl	1.00 ± 0.113	1.17 ± 0.135	
Urea (range, mg/dl)	18.0–43.6	24–51	0.0071^†^
Mean ± SD, mg/dl	32.2 ± 8.16	37 ± 7.18	

† Significant from control subjects at p < 0.05.

AIS: Acute ischemic stroke; Cr: Creatinine; HDL: High density lipoprotein; LDL: Low density lipoprotein; RBG: Random blood glucose; SD: Standard deviation; T.ch: Total cholesterol; TG: Triglyceride; VLDL: Very low density lipoprotein.

### Expression pattern of the studied miRNAs in AIS patients

**Figure 1.**
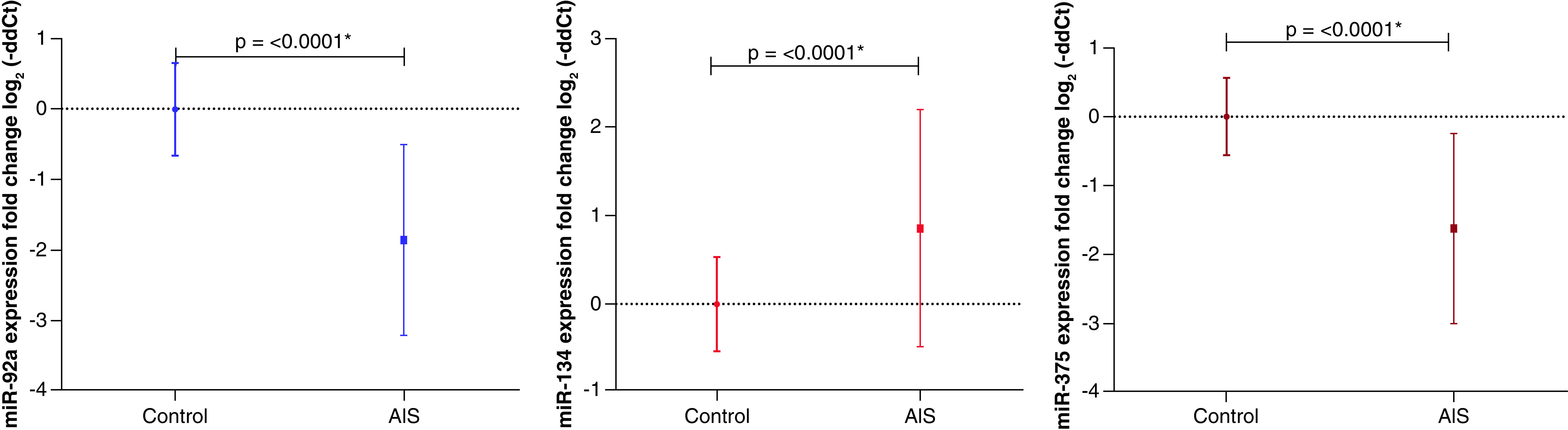
The expression pattern of the studied miRNAs in acute ischemic stroke patients.

The expression patterns of three miRNAs were studied in AIS patients' sera: miR-92a, miR-134, and miR-375. The downregulated miRNAs were miR-92a (68; 97.1%; -1.86 ± 1.36) and miR-375 (64; 91.4%; -1.63 ± 1.38). While miR-134 showed a predominant upregulated expression pattern (55; 78.6%; 0.853 ± 1.34). The most consistent expression pattern was observed with miR-92a (Figure 1 & Table 2).

**Table 2.** The expression pattern of serum-selected miRNAs in different groups.

MiRNAs	General (AIS) (n = 70)	DM (n = 15)	HTN (n = 25)	DM + HTN (n = 21)	Non DM + Non HTN (n = 9)
MiR-92a Mean ± SD Median (IQR) Range p	-1.86 ± 1.36 -2.62 to -0.934 -5.39 to 0.850 <0.0001^†^	-1.59 ± 1.09 -2.10 to -0.884 -3.80 to -0.032 0.0028^†^	-1.57 ± 1.30 -2.09 to -0.896 -4.85 to 0.850 0.0001^†^	-2.07 ± 1.50 -3.60 to -0.710 -4.20 to 0.191 <0.0001^†^	-2.79 ± 1.31 -3.31 to -1.88 -5.39 to -1.84 <0.0001^†^
MiR-134 Mean ± SD Median (IQR) Range p	0.853 ± 1.34 0.284 to 1.72 -3.13 to 2.98 <0.0001^†^	0.355 ± 1.11 -1.77 to 1.73 -0.671 to 1.04 0.9142	0.781 ± 1.54 -0.059 to 1.79 -3.13 to 2.63 0.1437	1.11 ± 1.60 0.659 to 1.70 -1.15 to 2.98 0.0212^†^	1.27 ± 1.67 0.144 to 2.48 -1.84 to 2.54 0.1234
MiR-375 Mean ± SD Median (IQR) Range p	-1.63 ± 1.38 -2.20 to -1.12 -5.64 to 2.74 <0.0001^†^	-2.00 ± 1.82 -5.64 to 1.46 -3.02 to -0.995 0.0002^†^	-1.51 ± 1.73 -2.42 to -0.606 -5.57 to 2.74 0.0002^†^	-1.60 ± 0.638 -2.14 to -1.31 -2.80 to -0.161 0.0005^†^	-1.47 ± 0.492 -1.94 to -1.08 -2.19 to -0.808 0.0693

† Significant from control subjects at p < 0.05.

AIS: Acute ischemic stroke; DM: Diabetes mellitus; HTN: Hypertension; IQR: interquartile range; miRNA: MicroRNA; SD: Standard deviation.

### Expression pattern of the studied miRNAs between the groups of the studied patients

Furthermore, based on the routine biochemical investigations and the associated co-morbidities of AIS patients such as diabetes and hypertension, the AIS patients were furtherly classified into 4 groups; Hypertensive diabetic, hypertensive non-diabetic, diabetic non-hypertensive and non-hypertensive non-diabetic patients to find out whether the expression pattern could reveal a further picture of the disease and its associated risk factors.

**Figure 2.**
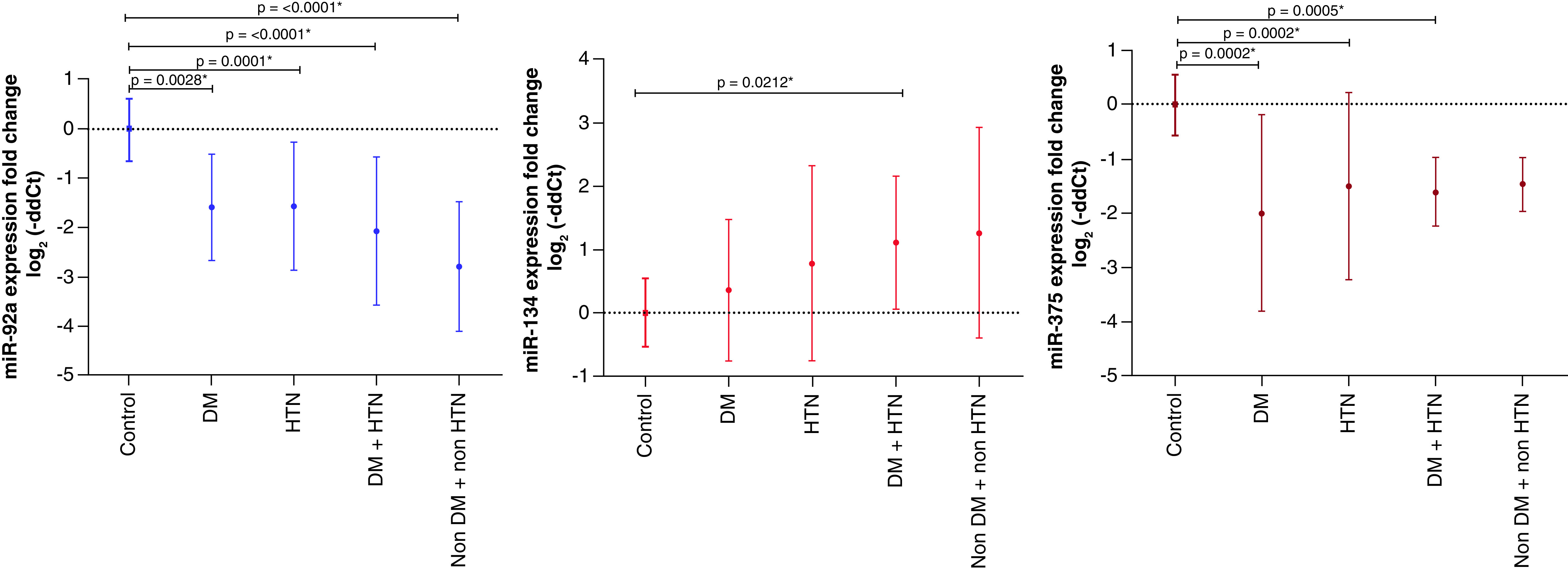
The expression pattern of the studied miRNAs among acute ischemic stroke groups. DM: Diabetes mellitus; HTN: Hypertension.

MicroRNA-92a showed the most consistent expression pattern in the 4 studied groups with significant differences. MiR-375 showed significant differences in 3 groups; diabetic non-hypertensive, hypertensive diabetic, and hypertensive non-diabetic, while miR-134 showed only a significant difference in hypertensive diabetics (Figure 2 & Table 2).

### The diagnostic accuracy of the studied miRNAs in AIS patients

**Figure 3.**
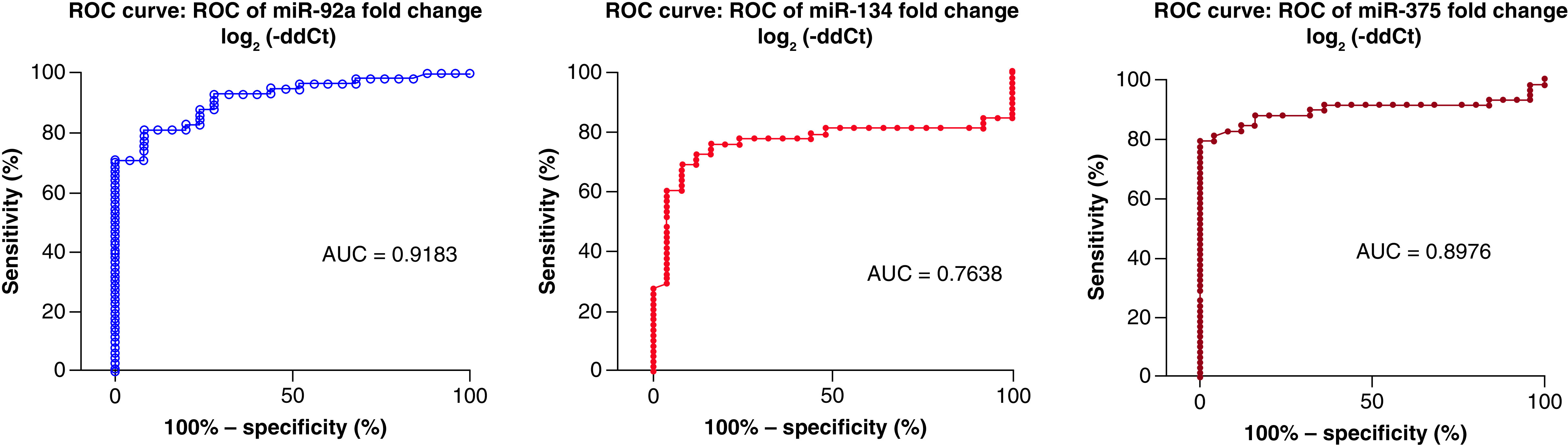
Receiver operator characteristic curve of the studied miRNAs. ROS: Receiver operator characteristic.

MicroRNA-92a and miR-375 revealed the best diagnostic accuracy for AIS diagnosis from control subjects (AUC = 0.9183 & AUC = 0.898, respectively) with a sensitivity of 81%, but with greater specificity for miR-375 (Sp = 96%), while the least diagnostic accuracy for diagnosis was found with miR-134 (AUC = 0.764 and Sn = 72.4%) (Figure 3 & Table 3).

**Table 3.** The diagnostic accuracy of the targeted miRNAs in AIS patients from the control subjects.

MiRNAs	Cutoff	AUC	Sn%	Sp%	95% CI	p-value
**MiR-92a**	<-0.821	0.9183	81%	92.0%	0.861–0.970	<0.0001
**MiR-134**	>0.4869	0.764	72.4%	88.0%	0.658–0.869	0.0001
**MiR-375**	<-0.8185	0.898	81.0%	96.0%	0.827–0.969	<0.0001

### Pearson's correlation coefficient between the studied miRNAs

Correlation between the expression of studied miR-92a, miR-134, and miR-375 in AIS patients with the clinicopathological findings was evaluated by Pearson's linear correlation coefficient (Table 4). A significant moderate strength positive correlation was found between miR-92a and miR-134 expression (r = -0.585 at p < 0.0001).

**Table 4.** Pearson correlation coefficient between the studied miRNAs and patients' characteristics.

Parameters	miR-92a		miR-134		miR-375	
	r	p-value	r	p-value	r	p-value
Age	0.0195	0.8844	0.180	0.1760	0.0812	0.5447
Rbs	-0.0499	0.7101	-0.0821	0.5399	-0.143	0.2859
T.ch	-0.0125	0.9259	0.137	0.3055	0.169	0.2054
Tg	-0.0508	0.7052	0.136	0.3093	0.0864	0.5190
LDL-ch	0.0301	0.8224	0.0949	0.4786	0.122	0.3631
HDL-ch	-0.113	0.3997	0.101	0.4501	0.128	0.3387
VLDL-ch	-0.0508	0.7052	0.136	0.3093	0.0864	0.5190
Cr	-0.200	0.1327	0.0912	0.4960	0.0279	0.8354
Urea	-0.228	0.0847	0.0753	0.5745	-0.204	0.1251
miR-92a	1	–	-0.585	<0.0001^†^	-0.228	0.0699
miR-134	-0.585	<0.0001^†^	1	–	0.111	0.3821
miR-375	-0.228	0.0699	0.111	0.3821	1	–

† Significant linear correlation at p < 0.05 (two-tailed).

Cr: Creatinine; HDL: High density lipoprotein; LDL: Low density lipoprotein; RBG: Random blood glucose; T.ch: Total cholesterol; TG: Triglyceride; VLDL: Very low density lipoprotein.

## Discussion

The diagnosis of AIS can only be done through contrast MRI and there are no current systemic biomarkers for early diagnosis. Circulating miRNAs have caught the research interest as potential candidate non-invasive markers due to their unique disease profile, stability, and reproducibility in serum and plasma [28]. This study aimed to investigate the expression patterns of miR-92a, miR-134, and miR-375 in AIS patients' sera and to explore their clinical potential in diagnosis of AIS.

MicroRNA-92a is a conserved endothelial cell-specific miRNA located in the miR-17approximately 92 cluster at chromosome 13q31.3. It is highly expressed in endothelial cells, mediating its communication with macrophages, thus contributing to atherosclerosis progression [29]. Many studies showed the significant oncogenic role and overexpression of miR-92a in different types of cancers like colorectal cancer, lung cancer, papillary thyroid carcinoma, and gastric cancer [15,30–32].

Regarding the expression pattern, miR-92a showed a predominant downregulation in AIS patients' sera with the most consistent expression pattern among all studied miRNAs. Also, it was downregulated in all groups in comparison to the control subjects. However, no significant difference was found between the patients' groups. And concerning diagnostic accuracy, miR-92a showed the best diagnostic potential and the highest diagnostic accuracy for AIS patients from control subjects (AUC = 0.9183, P = <0.0001) with Sn = 81% and Sp = 92% compared with the other four studied miRNAs.

It has been observed that MicroRNA-92a inhibition plays a role in improving angiogenesis and recovery in murine models with chronic ligation of the anterior descending coronary artery, critical limb ischemia, vascular injury, and in a porcine model of ischemia/reperfusion injury [33]. The regulation of angiogenesis by miR-92a occurs by targeting several pro-angiogenic proteins, including the integrin subunit α5 (ITGA5) and Sirtuin (SIRT1) [20,34].

In the present study, miR-92a also showed a significant difference in the group of AIS with hypertension. Interestingly, our results were promising with a previous study reporting that circulating miR-92a may act as a potential noninvasive atherosclerosis marker in essential hypertensive patients [35]. In addition, miR-92a showed a significant difference in diabetes mellitus patients with AIS from the control group. Previously, it was studied that the expression of miR-92a was increased in patients having both diabetes and acute coronary syndrome [36]. Others found that miR-92a with diabetes could inhibit apoptosis induced by a high-glucose environment and could increase insulin secretion by targeting Kruppel Like Factor 2 (KLF2) [37]. Therefore, miR-92a may have an apoptotic role based on the previous findings.

MicroRNA-134 is a brain-specific miRNA that is associated with the development of the dendritic and synaptic spines and belongs to chromosome 14q32 miRNA clusters [38,39]. Regarding the expression pattern, it was upregulated with a significant difference from the control group as well as significant upregulation in hypertensive diabetic versus control subjects with no significant difference between each group and others. Considering its diagnostic value and ROC curve analysis, miR-134 showed good diagnostic accuracy among the studied miRNAs (AUC = 0.764; p = 0.0001) with Sn = 72.4% and Sp = 88%.

In a previous study, it was reported that miR-134 was downregulated in ischemic neurons, which might influence neuronal cells against ischemic injury apoptosis through enhancing Heat Shock Protein Family A (HSPA12B) protein levels, by targeting the 3′ UTR of HSPA12B leading to neural cell death and apoptosis [40]. Moreover, our results were promising and in agreement with the results of Zhou *et al.*, who observed that there was a significant increase of exosomal miR-134 in AIS patients within 24 h after stroke onset when compared with that of the control group, suggesting that it can be a possible potential biomarker to differentiate between AIS patients and non-stroke subjects [41]. Interestingly, one study observed a neuronal cell protective effect against death upon downregulation of miR-134 [42].

MicroRNA-375 was first identified by Poy *et al.*, in 2004 as a specific miRNA for the pancreatic β-cells and has a role in the regulation of insulin secretion [22]. This was the first time to investigate miR-375 expression patterns in AIS patients' sera. Human miR-375 is encoded by the chromosomal region 2q35 [43].

Regarding the expression pattern, it was downregulated with a significant difference from the control group and significant downregulation in 3 groups: diabetic non-hypertensive, hypertensive diabetic, and hypertensive non-diabetic versus control subjects with no significant difference between each group and others. Considering its diagnostic value and ROC curve analysis, miR-375 showed the second-greatest diagnostic accuracy among the studied miRNAs (AUC = 0.898, p = <0.0001) with Sn = 81% and Sp = 96%.

Our results agreed with the findings of Ou *et al.*, who found down-regulation of miR-375 in the rat I/R (ischemia/reperfusion) brain suppressed apoptosis caused by I/R injury through the binding to3`-UTR region of connective tissue growth factor (CTGF) target gene mRNA. In addition, it showed that miR-375/CTGF mediated protective effects are associated with p21/PI3K/Akt signaling pathways, which are cross-reactive with insulin receptors signaling [44].

The association of downregulated circulating miR-375 levels with myocardial infarction (MI) has been studied before [45]. A further study identified PIK3CA, MAPK3, PAFAH1B1, RHOA, ERBB2, MYC, PRKCA, CTNNB1 and CDC42 as crucial genes in the miR-375 regulated network and predicted the possible function of miR-375 in the heart muscle, consisting mainly in the regulation of the Rho-GTPases-dependent signaling pathways, therefore, miR-375 may function as a regulator for apoptosis in the heart muscle relaying on Rho-GTPases-dependent pathways [46].

In the present study, miR-375 also showed a significant difference in the group of AIS with diabetes. Previously, it was observed that miR-375 has a role in diabetes, where its mechanism depends on controlling the expression of myotrophin (MTPN) and phosphoinositide-dependent protein kinase-1 (Pdk1) genes [47]. Another study confirmed that miR-375 may have an effective role in the regulation of beta-cell glucose metabolism and insulin secretion [22]. MiR-375 also showed, in our study, a significant difference in the hypertensive subgroup versus control subjects, which agrees with a previous study [48].

The miR-375 target genes may disclose the potential mechanisms and metabolic role of miR-375 in AIS and highlight its potential value as a novel diagnostic marker in human acute ischemic stroke, particularly with diabetes.

There are some limitations to the present study; first, because it was designed as a pilot study, the selected subjects' sample size was not large sufficiently. So, a study on larger cohorts is recommended for further validation of these results.

Second, the selection of the studied miRNAs was dependent on the literature and the expected mechanisms to be involved in AIS, without previous miRNAs screening which may show other predominant miRNAs in the Egyptian population. So, wide screening of miRNAs is recommended in future studies.

Third, it was a retrospective pilot model on affected persons without previous detection of these miRNAs before being affected by AIS to explore their expression pattern before the AIS.

Fourth, patients' medications and body weight weren't documented in this study.

Finally, our results express the immediate change in miRNAs together with the onset of AIS regardless of the correlation with long-term disabilities or comorbidities secondary to AIS (prognostic value).

## Conclusion

The present study's findings showed that there is a potential role for the studied miRNAs in AIS especially miR-92a and miR-375 which may be considered promising diagnostic biomarkers. In addition, miR-375 was related to diabetes so can be one of the screening tools for risk prediction of AIS with diabetes.

**Summary points**

- MicroRNAs are endogenous, small, non-coding, single-stranded RNA molecules with great serum stability and easier detection.
- We hypothesized the implication of microRNAs 92a, 134, and 375 in the pathogenesis of acute ischemic stroke with subsequent changes in their circulating levels.
- Serum miR-92a and miR-375 were predominantly down-expressed, while miR-134 was upregulated.
- Serum miR-92a and miR-375 showed the highest diagnostic accuracy, while miR-375 has the highest specificity.
- Both miR-92a and miR-375 serum levels may be promising biomarkers for the early detection of acute ischemic stroke.
